# Resource-efficient retrieval-augmented question answering for the Indian Lok Sabha dataset

**DOI:** 10.3389/frai.2026.1798021

**Published:** 2026-06-16

**Authors:** S. Sivakumar, S. P. Meenakshi

**Affiliations:** 1 Department of Mathematics, School of Advanced Sciences, Vellore Institute of Technology, Vellore, India; 2 Department of Database Systems, School of Computer Science and Engineering, Vellore Institute of Technology, Vellore, India

**Keywords:** DistilGPT-2, Indian Lok Sabha, natural language processing, question answering system, retrieval-augmented generation

## Abstract

**Introduction:**

The Indian Lok Sabha generates a continuously expanding corpus of legislative records, predominantly archived as unstructured PDF files. Effective public access remains limited due to the shortcomings of keyword-based retrieval systems and the hallucination risks of general-purpose Large Language Models (LLMs).

**Methods:**

This paper presents a domain-specific, resource-efficient Retrieval-Augmented Generation (RAG) framework employing DistilGPT-2 (82M parameters) as the generative model, grounded via FAISS-based semantic retrieval using Sentence-BERT embeddings. The pipeline integrates multi-stage PDF preprocessing, semantic indexing, and context-aware response generation. Evaluation was conducted on 450 queries spanning simple, complex, and compound categories, assessed by human annotators using factual accuracy and a five-point relevance scale.

**Results:**

The proposed RAG + DistilGPT-2 framework achieves 94% factual accuracy and a relevance score of 4.6 out of 5, substantially outperforming zero-shot baselines (80% factual accuracy without RAG), while maintaining an average end-to-end inference latency of 1,800 milliseconds (ms) on standard CPU hardware.

**Discussion:**

The results demonstrate that combining domain-specific retrieval with a lightweight generative model effectively mitigates hallucination and reduces computational overhead, offering a scalable, transparent solution for e-governance applications without reliance on GPU infrastructure.

## 1 Introduction

The proliferation of digital governance initiatives worldwide has resulted in an unprecedented accumulation of legislative data. In the context of the world's largest democracy, the Indian Parliament (Lok Sabha) generates a vast corpus of official records, including parliamentary debates, starred and unstarred questions, legislative bills, and standing committee reports. These documents constitute the primary repository of legislative intent, policy formulation, and procedural governance (Karpukhin et al., 2020). However, the sheer volume and largely unstructured nature of these records, predominantly archived as static PDF files, create a significant information bottleneck. For citizens, policy analysts, and journalists, navigating this data remains a labor-intensive task, often constrained by traditional keyword-based retrieval systems that lack semantic understanding and fail to capture contextual nuances (Devlin et al., 2019).

Natural Language Processing (NLP) has witnessed rapid advancements with the emergence of LLMs such as GPT-4 and LLaMA-2 (Abercrombie and Batista-Navarro, 2020), which demonstrate strong capabilities in language understanding and text generation. Despite their success in open-domain applications, the application of general-purpose LLMs to legal and parliamentary domains introduces critical challenges.

General-purpose LLMs are trained on large-scale, open-domain corpora that include heterogeneous and non-authoritative sources. When queried about specific legislative procedures or historical parliamentary debates, these models are prone to hallucinations, producing fluent but factually incorrect responses (Ji et al., 2023). In a parliamentary setting, where accuracy and verifiability are essential, such inconsistencies significantly undermine trust and usability. State-of-the-art LLMs require substantial computational resources, typically relying on high-end GPUs for efficient inference (Vaswani et al., 2017). These requirements limit their feasibility for deployment in resource-constrained public sector environments and lightweight web-based governance platforms.

To mitigate these limitations, this study proposes a domain-specific and resource-efficient conversational agent tailored exclusively to the Lok Sabha domain. The proposed system adopts a RAG framework (Lewis et al., 2020), which decouples knowledge storage from language generation. Instead of relying solely on the parametric memory of a language model, relevant contextual information is dynamically retrieved from an indexed corpus of authenticated Lok Sabha documents and provided as input to the generator, thereby improving factual grounding and response reliability.

A central contribution of this work is the strategic selection of DistilGPT-2 as the core generative model (Touvron et al., 2023). In the current landscape of Natural Language Processing, the dominant trend has been to scale model parameters into the billions (e.g., GPT-4, LLaMA) to achieve marginal performance gains. However, this trend, often termed “Red AI,” necessitates substantial computational resources, high energy consumption, and specialized GPU infrastructure, rendering such models impractical for resource-constrained public sector deployments. In contrast, DistilGPT-2 represents a distilled variant of the GPT-2 architecture, developed using knowledge distillation techniques where a smaller “student” model is trained to reproduce the behavior of a larger “teacher” model. With approximately 82 million parameters, DistilGPT-2 is significantly lighter than the 1.5 billion parameters of GPT-2 XL (Brown et al., 2020). This reduction in size translates to lower memory requirements and faster inference speeds, validating the feasibility of deploying the system on standard commodity hardware.

### 1.1 Motivation

The adoption of Artificial Intelligence (AI) in public-sector governance is increasingly constrained by the high computational and energy demands of state-of-the-art LLM, which typically require specialized GPU infrastructure for efficient deployment. While such models exhibit strong generative capabilities, their operational costs, latency, and scalability challenges render them impractical for resource-constrained e-governance platforms, such as public-facing parliamentary information systems.

To address these challenges, this study proposes a resource-efficient RAG framework. The system employs DistilGPT-2 a distilled generative model with approximately 82 million parameters thereby significantly reducing memory footprint and inference complexity. Crucially, instead of embedding factual knowledge within the model's parameters, authoritative legislative content is externalized into a dense vector index implemented using FAISS, optimized for CPU-based similarity search. This decoupling of retrieval and generation facilitates efficient indexing and querying of large parliamentary corpora while maintaining deployability on commodity hardware.

### 1.2 Contributions

The main contributions of this work are threefold:

1. A resource-efficient Retrieval-Augmented Generation framework specifically designed for Indian Lok Sabha parliamentary question answering.
2. A systematic component selection strategy ensuring low-latency CPU deployment. This includes the use of MiniLM embeddings, FAISS-based semantic retrieval, DistilGPT-2 as the lightweight generator, LangChain for modular pipeline orchestration, and an effective text chunking strategy.
3. Investigation of lightweight models, when grounded through retrieval, can achieve high factual accuracy while reducing hallucination, eliminating the need for large GPU-based LLMs.
4. Design of a metric-driven assessment framework that evaluates response quality and efficiency through relevance scoring, factual verification, and latency measurements.

## 2 Related work

The development of automated systems for parliamentary monitoring and legal information retrieval lies at the intersection of NLP, Information Retrieval (IR), and Artificial Intelligence for governance. This section reviews relevant research across these domains and identifies the limitations that motivate the proposed lightweight, retrieval-augmented conversational system for the Lok Sabha.

Early approaches to accessing legal and parliamentary documents primarily relied on traditional IR techniques such as Boolean retrieval and TF-IDF weighting schemes. Although effective for exact keyword matching, these methods suffer from the semantic gap, defined as the mismatch between user-formulated natural language queries and the formal, domain-specific terminology used in legislative texts (Zhong et al., 2020). Recent studies have adopted neural language models to address this limitation. (Chalkidis et al. 2020) demonstrated that domain-adapted models such as Legal-BERT (Reimers and Gurevych, 2019) significantly outperform general-purpose models on legal classification, information extraction, and named entity recognition tasks. In parliamentary contexts, NLP techniques have been applied to topic modeling, sentiment analysis, and discourse analysis to study political polarization and legislative behavior (Abercrombie and Batista-Navarro, 2020). However, these efforts largely focus on analytical insights rather than interactive question answering. Conversational systems that allow citizens to query parliamentary proceedings in natural language remain limited, particularly for the Indian Lok Sabha, which presents unique procedural language and document structures.

LLMs such as GPT-3, PaLM, and LLaMA-2 have achieved strong performance in open-domain question answering through zero-shot and few-shot learning (Johnson et al., 2019). Despite these advances, their deployment in high-stakes domains such as governance is constrained by hallucination, wherein models generate fluent but factually incorrect or unverifiable content (Ji et al., 2023). In parliamentary applications, factual inaccuracies such as conflating legislative bills or inventing procedural precedents are unacceptable. Additionally, general-purpose LLMs are limited by a fixed knowledge cutoff, preventing access to newly published parliamentary debates and documents without retraining. These characteristics significantly reduce their suitability for dynamic legislative environments like the Lok Sabha.

RAG, introduced by (Lewis et al. 2020), addresses hallucination and knowledge obsolescence by integrating document retrieval with text generation. RAG architectures combine a parametric language model with a non-parametric memory, typically implemented as a dense vector index (Gao et al., 2020). At inference time, relevant document segments are retrieved and injected into the model's input context, ensuring responses are grounded in authoritative sources. RAG-based systems have shown promising results in legal question answering and case law retrieval, outperforming purely generative approaches in terms of factual consistency.

Recent advances in retrieval-augmented generation have incorporated more powerful encoder-decoder and instruction-tuned architectures such as FLAN-T5 (Chung et al., 2022) and similar transformer-based models, which demonstrate strong performance in question answering and reasoning tasks. Instruction-tuned models such as FLAN-T5 have demonstrated strong performance in reasoning-intensive question answering tasks. However, their deployment in real-world systems is often constrained by computational cost, motivating the use of lightweight retrieval-augmented architectures in resource-limited environments. Recent studies have explored the use of transformer-based architectures for handling complex and long-context question answering tasks. For instance, (Sivakumar and Meenakshi 2025a) proposed a BERT-based framework for modeling and analyzing question answering systems capable of handling long-context inputs and compound queries. Their work demonstrates the effectiveness of deep contextual embeddings in capturing semantic relationships across extended textual inputs, particularly in scenarios requiring multi-step reasoning. However, such models typically require substantial computational resources, including GPU acceleration, which may limit their applicability in resource-constrained public-sector environments. In contrast, the present study focuses on a lightweight, CPU-deployable architecture, prioritizing efficiency and accessibility over large-scale model capacity (Schwartz et al., 2020).

The Green AI paradigm emphasizes the development of computationally efficient and environmentally sustainable machine learning systems (Schwartz et al., 2020). Knowledge distillation has emerged as a key technique in this context, enabling smaller student models to retain much of the performance of larger teacher models while significantly reducing computational cost (Sanh et al., 2019). DistilGPT-2 represents a distilled variant of GPT-2 that offers faster inference and a smaller memory footprint. While smaller models are often perceived as less capable of complex reasoning, this limitation can be mitigated in retrieval-augmented settings. By delegating factual knowledge storage to the retrieval component, the generative model primarily focuses on synthesis and response formulation rather than memorization.

Table 1 presents a comparative analysis of existing approaches in legal and parliamentary natural language processing against the proposed framework. Traditional information retrieval techniques based on Boolean matching and TF-IDF remain computationally efficient but are fundamentally limited by their inability to capture semantic context or support natural language querying (Zhong et al., 2020). Domain-adapted legal and parliamentary NLP models, such as Legal-BERT, demonstrate strong performance in analytical tasks including classification, named entity recognition, and sentiment analysis; however, these systems are primarily designed for monitoring and trend analysis rather than interactive question answering for citizens (Chalkidis et al., 2020).

**Table 1:** Comparison of existing approaches in legal/parliamentary NLP with the proposed framework.

Approach category	Core technique	Key advantages	Identified limitations
Traditional IR	Boolean retrieval, TF-IDF	Computationally inexpensive; effective for exact keyword matching.	Suffers from the “semantic gap”; lacks contextual understanding for natural language queries (Zhong et al., 2020).
**Legal & parliamentary NLP**	Domain-adapted models (e.g., legal-BERT), topic modeling	High accuracy on domain-specific tasks (e.g., NER, classification, and sentiment analysis; Chalkidis et al., 2020).	Designed for analysis rather than interaction; lacks conversational QA capabilities for end-users.
**General large language models**	Zero-shot learning (e.g., GPT-4, LLaMA)	Strong open-domain reasoning and fluent text generation.	Prone to hallucinations (Ji et al., 2023); static knowledge cutoff; prohibitive computational and energy costs.
**Standard RAG systems**	Dense retrieval + large generative models (e.g., GPT-3)	Reduces hallucinations by grounding responses in retrieved evidence (Lewis et al., 2020).	High inference latency and operational costs; often impractical for resource-constrained public-sector deployment.
**Proposed framework**	**RAG + DistilGPT-2 + FAISS**	**Factually grounded responses; low-latency inference on commodity hardware; specifically designed for unstructured Lok Sabha PDFs**.	**Performance bounded by the smaller generator's fluency; requires accurate retrieval for high-quality outputs**.

Recent general-purpose large language models offer strong zero-shot reasoning and linguistic fluency but are susceptible to hallucinations, constrained by static knowledge cutoffs, and require substantial computational resources, which limits their suitability for public-sector deployment (Ji et al., 2023). Recent work by (Sivakumar and Meenakshi 2025b) focuses on efficient data preprocessing techniques for extractive question answering models. Their study highlights the importance of structured preprocessing pipelines in improving model performance, particularly for handling long-context inputs and compound queries. The proposed methods enhance data quality and retrieval effectiveness, which are critical for downstream question answering tasks. Retrieval-augmented generation systems partially mitigate hallucination by grounding responses in external documents, yet they often rely on large generator models that incur high inference latency and operational costs (Lewis et al., 2020). In contrast, the proposed framework integrates a lightweight DistilGPT-2 model within a retrieval-augmented generation pipeline (Hu et al., 2021), achieving a balance between factual accuracy and computational efficiency while being specifically optimized for unstructured Lok Sabha parliamentary documents.

## 3 Methodology

**Figure 1:**
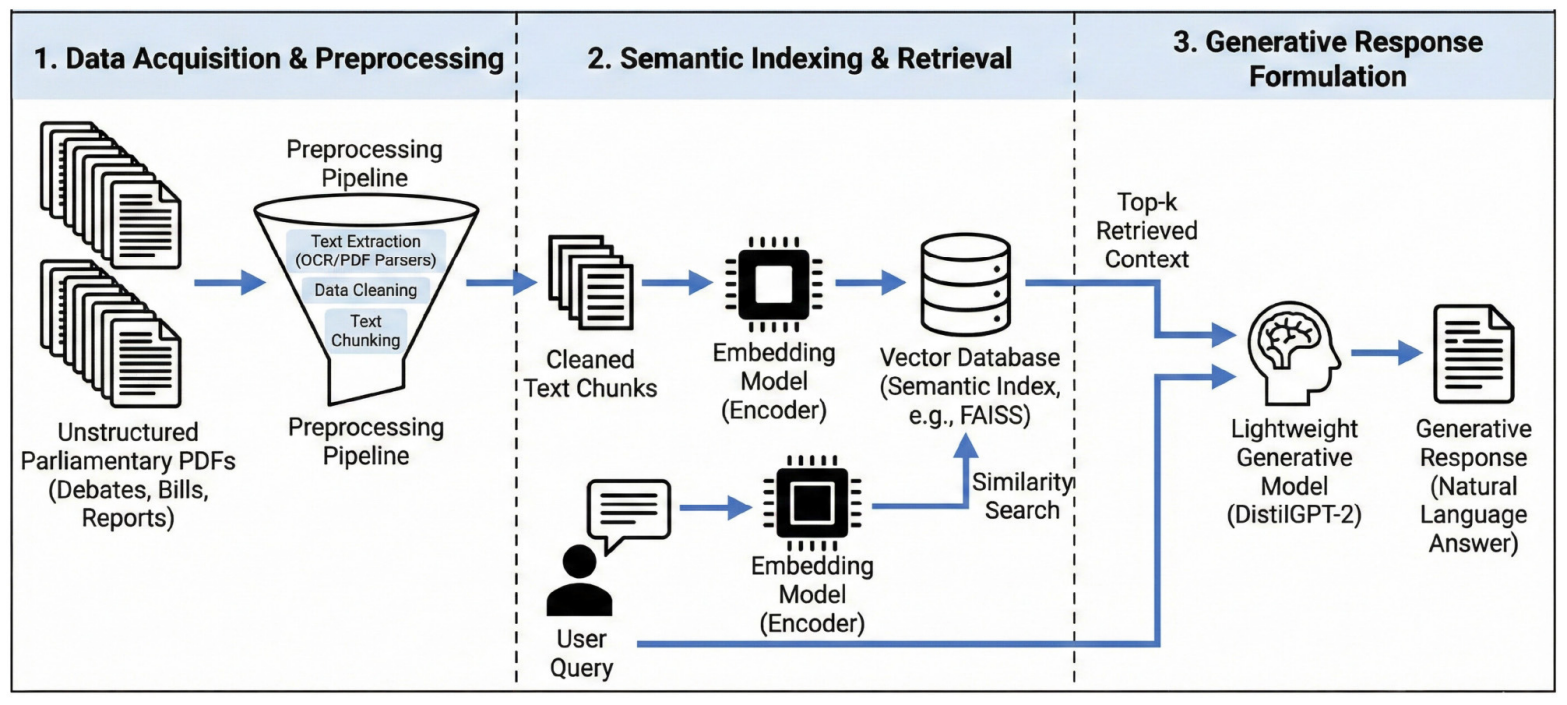
Architecture of the proposed RAG framework with DistilGPT-2 for Lok Sabha question answering. The pipeline comprises data preprocessing, FAISS-based retrieval, and lightweight generative response formulation.

This section details the technical framework and implementation of the proposed parliamentary question-answering system. The methodology is structured as a three-stage pipeline: (1) domain-specific data acquisition and preprocessing, (2) semantic indexing and retrieval of parliamentary content, and (3) response generation via a lightweight, retrieval-augmented language model. The core innovation lies in the strategic integration of DistilGPT-2 within a Retrieval-Augmented Generation (RAG) architecture, optimized for computational efficiency and factual consistency. This design prioritizes deployment on commodity hardware while maintaining robust performance on domain-specific parliamentary queries. The following subsections elaborate on each component, with particular emphasis on the suitability of DistilGPT-2 for synthesizing context-aware responses from unstructured Lok Sabha proceedings. Figure 1 illustrates the complete system architecture. In this study, efficiency is evaluated in terms of computational latency and CPU-based deployment. Direct measurement of energy consumption is not performed. The proposed system is intended to enhance computational efficiency through lightweight model architecture and CPU-based deployment, with efficiency assessed primarily through inference latency rather than direct energy measurement.

### 3.1 Data collection and corpus construction

The primary data source comprises digitized parliamentary proceedings obtained from the official repository of the Indian Parliament (Sansad Lok Sabha Questions Repository). To improve dataset generalizability, this study incorporates parliamentary documents from multiple legislative periods, including 2021–2022, 2022–2023, and 2023–2024 (17th Lok Sabha). The most recent sessions, spanning from the Monsoon Session 2023 to the Budget Session 2024, were used as the primary benchmark period due to their policy relevance and data completeness. The collected corpus includes 3,500 PDF documents covering debates, starred and unstarred questions, and bill discussions, totaling approximately 850,000 sentences. These documents capture a wide range of contemporary governance issues, legislative amendments, and ministerial responses, ensuring strong relevance to real-world parliamentary discourse.

To ensure systematic data acquisition, a dedicated web scraper with politeness delays was employed, followed by integrity checks to verify document completeness. The data were organized chronologically and categorized by document type (Debates, Q&A, Bills) to support both temporal and thematic retrieval. Importantly, the dataset spans multiple policy domains, including economic policy, agriculture, technology, and public administration. This diversity ensures that the corpus is not restricted to a single topic or narrow domain, but instead reflects the heterogeneous nature of parliamentary proceedings.

For evaluation purposes, queries were categorized into three types based on complexity: (1) simple queries requiring direct factual retrieval, (2) complex queries requiring contextual understanding and reasoning, and (3) compound queries involving multiple conditions or multi-step reasoning. Each parliamentary document contains multiple question-answer pairs (typically 3–6 per document), with answers available in diverse formats such as descriptive text, tabular data, and detailed explanations.

This study focuses exclusively on Lok Sabha proceedings and does not incorporate Rajya Sabha data. The objective is to design and evaluate a resource-efficient question answering system within a well-defined parliamentary domain, rather than to model the entirety of the Indian Parliament.

The dataset, while limited to selected Lok Sabha sessions across 2021–2024, captures diverse parliamentary activities across multiple policy sectors, ensuring variability in linguistic patterns, query structures, and contextual complexity. Accordingly, it serves as a representative corpus for domain-specific system evaluation.

### 3.2 Data preprocessing pipeline

**Figure 2:**
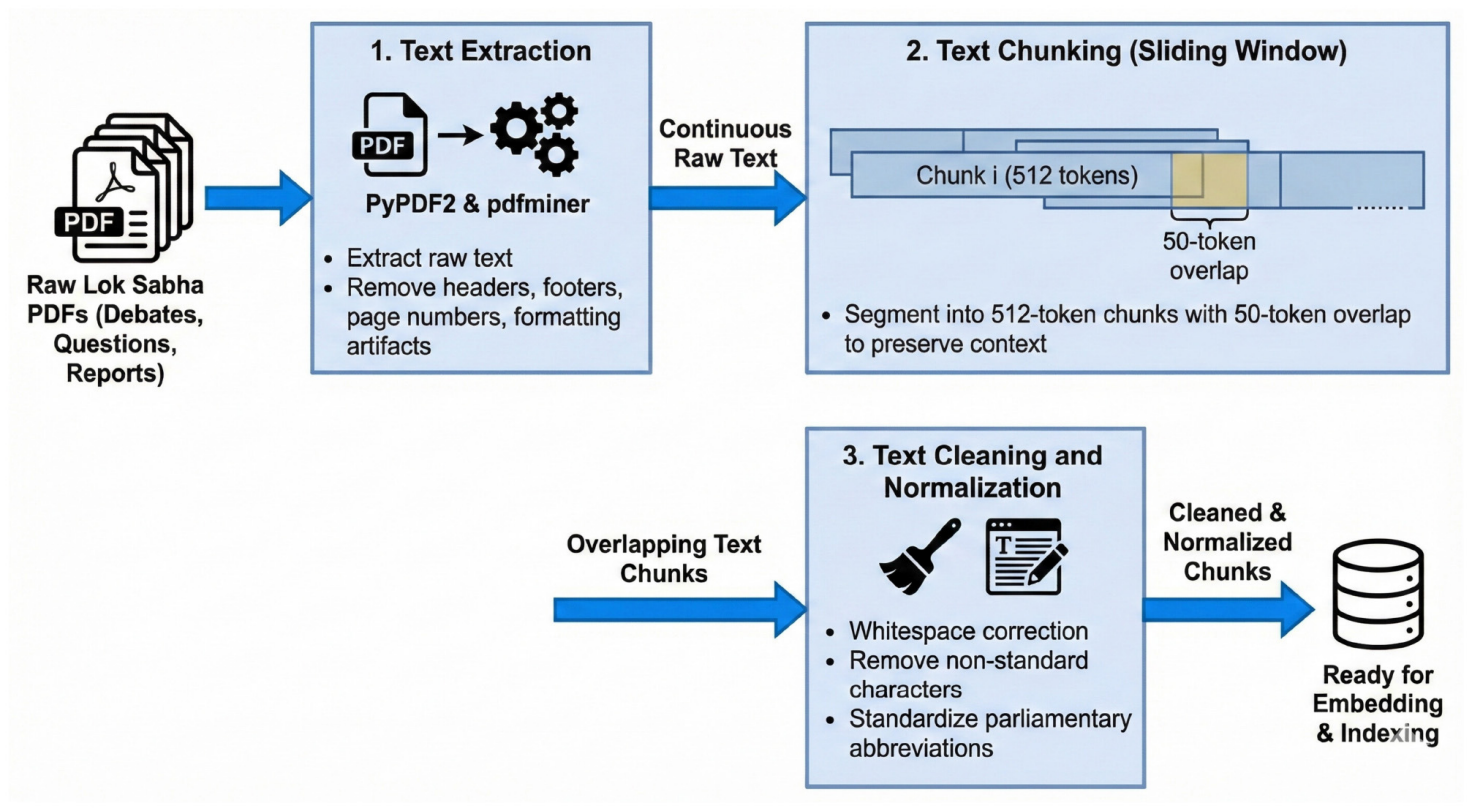
Multi-stage preprocessing pipeline for converting unstructured Lok Sabha PDFs into a clean, indexed corpus.

The raw PDF documents undergo a multi-stage preprocessing pipeline to transform unstructured parliamentary text into a clean, machine-readable format suitable for indexing and retrieval. First, Optical Character Recognition (OCR) using Tesseract OCR is applied to extract raw text, with post-processing to correct common OCR errors in parliamentary terminology. The text is then cleaned through a rule-based pipeline that removes headers, footers, page numbers, and procedural boilerplate (e.g., “Honorable Speaker,” “laid on the Table”). Subsequently, the text is segmented into coherent semantic units-each corresponding to a complete speaker turn, question, or answer-using a combination of parliamentary-specific heuristics (e.g., speaker tags like “SHRI...:”, question numbers) and layout cues. Named Entity Recognition (NER) is performed using a fine-tuned Legal-BERT model to identify and tag MPs, ministries, bills, dates, and locations. Finally, the processed text is chunked into overlapping passages of 256–512 tokens, optimized for subsequent embedding generation while preserving contextual continuity. Since PDF files are optimized for visual presentation rather than structured text processing, a dedicated preprocessing pipeline is required. The overall process is illustrated in Figure 2.

### 3.3 Semantic indexing and retrieval engine

The preprocessed text chunks are converted into dense vector representations to enable semantic search. We employ the “all-MiniLM-L6-v2” sentence transformer model (Reimers and Gurevych, 2019), which provides a strong balance between embedding quality and computational efficiency, with an output dimension of 384. Each chunk is encoded into a fixed-length vector representation capturing its semantic content. All embeddings are L2-normalized prior to indexing to ensure consistency in similarity computation. The embeddings are indexed using FAISS (Facebook AI Similarity Search) (Johnson et al., 2019) with the IndexFlatIP (inner product) configuration. This setup enables efficient cosine similarity search and is optimized for CPU-based retrieval.

During query time, a user's natural language query is encoded using the same embedding model, ensuring representation consistency. The system retrieves the top-*k* most relevant passages (empirically set to *k* = 3) based on similarity scores. The FAISS index is maintained in memory to minimize retrieval latency and support real-time inference.

**Figure 3:**
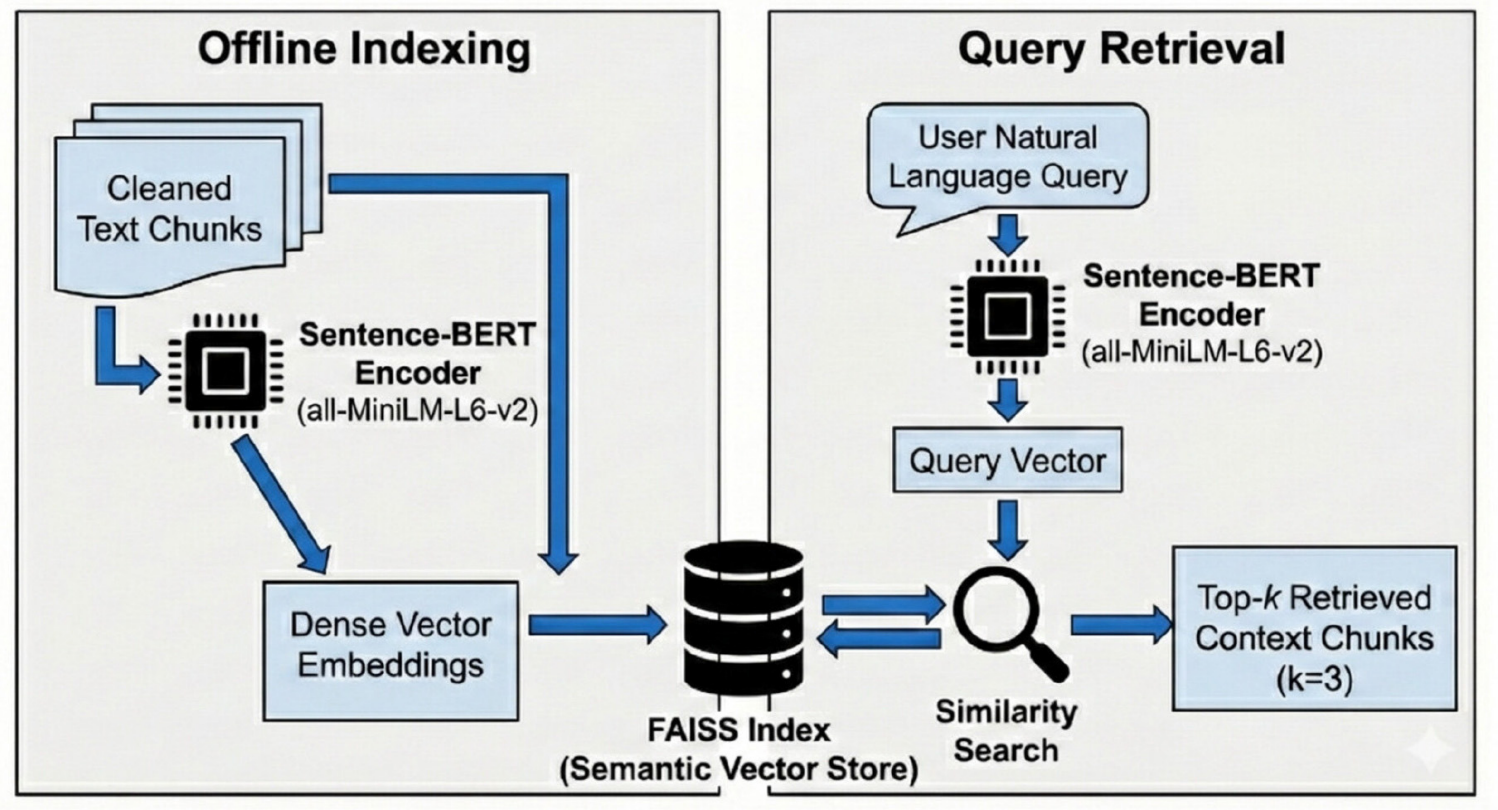
Semantic indexing and retrieval architecture. The pipeline encodes text chunks into embeddings, indexes them using FAISS, and retrieves relevant passages based on query similarity.

This retrieval-first design ensures that generated responses are grounded in the most relevant and authoritative parliamentary documents, significantly reducing hallucination while maintaining low-latency performance. The selected configuration balances retrieval accuracy and computational efficiency, making it suitable for resource-constrained environments. The overall semantic indexing and retrieval pipeline is illustrated in Figure 3.

### 3.4 Generative response formulation

**Figure 4:**
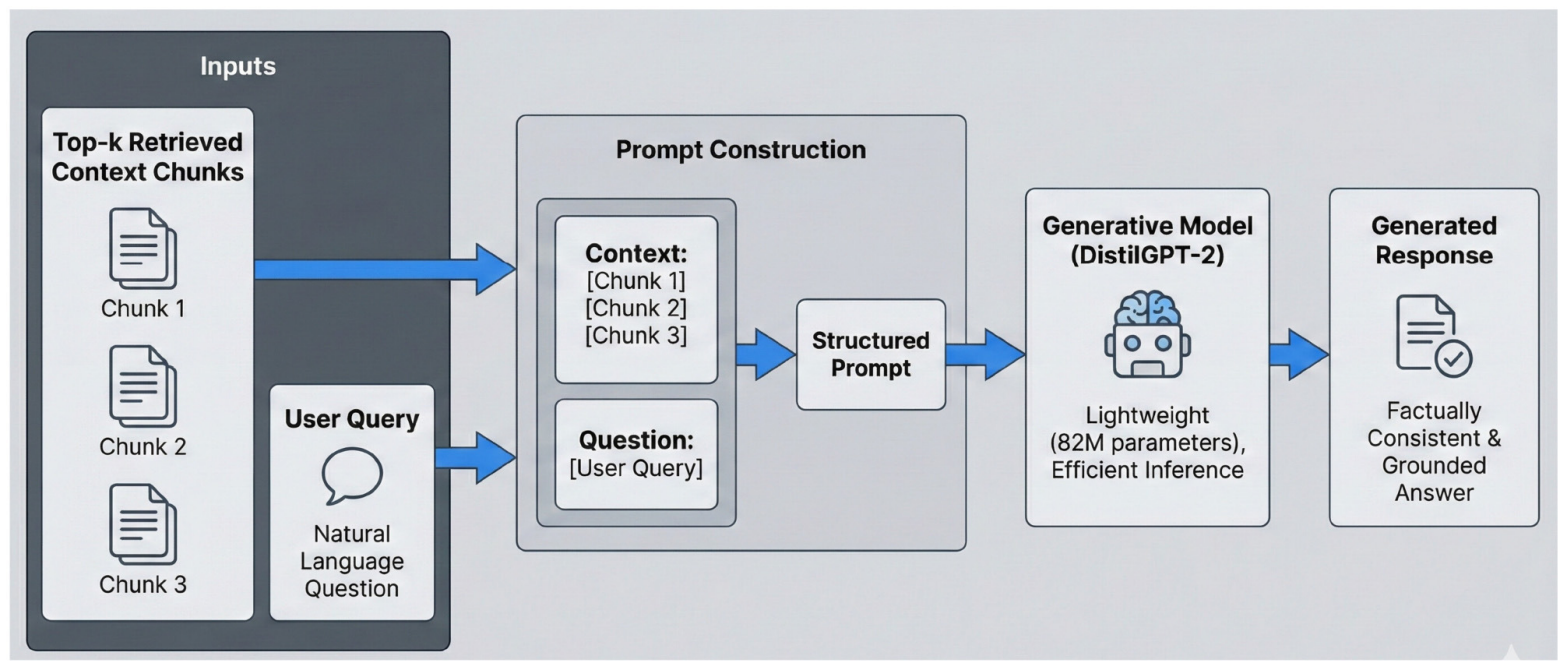
Generative response formulation workflow: retrieved passages are formatted into a structured prompt, processed by DistilGPT-2, and post-processed to produce a final parliamentary response.

The retrieved document chunks serve as grounded context for the generative model, ensuring that responses are anchored in authoritative parliamentary sources and substantially reducing hallucination risks. Figure 4 illustrates the complete workflow.

The top-*k* retrieved passages are concatenated into a structured prompt template designed to maximize factual accuracy and contextual relevance: You are a parliamentary assistant. Answer the user's question based ONLY on the provided context from the Lok Sabha proceedings. If the context does not contain sufficient information, respond with “The provided parliamentary records do not contain specific information on this query.”

Context: [Retrieved passage 1] [Retrieved passage 2] [Retrieved passage 3]

Question: [User query]

This template explicitly conditions the model to: (1) rely exclusively on provided evidence, (2) adopt a formal, parliamentary tone, and (3) acknowledge uncertainty when evidence is insufficient. The retrieved passages are truncated to fit within the model's maximum context window of 1024 tokens.

#### 3.4.1 Inference with DistilGPT-2

We employ DistilGPT-2 for response generation. The distilled architecture (82M parameters) provides an optimal balance between linguistic fluency and computational efficiency. During inference, we use nucleus sampling (top-*p* = 0.9) with temperature *T* = 0.7 to generate diverse yet coherent responses. The decoding process continues until an end-of-sequence token is produced or a maximum of 150 tokens is reached. To further enhance factual consistency, we implement a simple contextual verification step that checks for direct lexical overlap between key entities in the generated response and the retrieved passages.

#### 3.4.2 Post-processing and output formatting

Generated responses undergo post-processing to improve readability and parliamentary appropriateness. This includes:

- Formatting of parliamentary references (e.g., “as per the debate on 15th March 2024”).
- Standardization of MP titles and ministry names using the NER tags from preprocessing.
- Removal of repetitive or contradictory phrases through a simple redundancy filter.

The final output is structured to include both the synthesized answer and, when applicable, references to the source documents (session date, question number) for traceability.

#### 3.4.3 Efficiency considerations

The entire generation pipeline operates efficiently on CPU-only hardware. On a system with an Intel Xeon E5-2680 v4 processor (2.4 GHz), the average end-to-end latency, including document retrieval, prompt construction, and response generation, is 1,800 ms per query, meeting real-time interaction requirements for public-facing applications. All latency values reported in this study correspond to the complete end-to-end processing time.

#### 3.4.4 Implementation details and reproducibility

The key implementation settings used in the proposed system are summarized in Table 2.

**Table 2:** Implementation details and reproducibility settings.

Component	Configuration
Text preprocessing	PDF documents converted to text and segmented into chunks of 256–512 tokens with 15% overlap to preserve contextual continuity.
Embedding model	all-MiniLM-L6-v2 (384-dimensional embeddings); all vectors are L2-normalized.
FAISS index	IndexFlatIP (inner product similarity); cosine similarity achieved via normalized embeddings; index stored in-memory.
Retrieval parameters	Top-*k* = 3 most relevant chunks retrieved per query based on similarity scores.
Generation model	DistilGPT-2 with maximum input length of 512 tokens.
Decoding strategy	Greedy decoding with temperature = 0.7.
Hardware setup	CPU-only execution on Intel Xeon E5-2680 v4 (2.4 GHz), no GPU acceleration.

The implementation of the proposed retrieval-augmented question answering system is publicly available at: https://github.com/SivakumarS/LokSabha-RAG-QA-System The repository includes the complete pipeline for PDF preprocessing, semantic retrieval using FAISS, and response generation using DistilGPT-2, along with instructions for reproducing the experimental results.

### 3.5 Component selection rationale

Table 3 provides a comparative overview of the design choices for the core components in the proposed parliamentary RAG framework.

**Table 3:** Component selection and rationale for the proposed RAG framework.

Component	Selected option	Rationale	Alternative
Embedding model	all-MiniLM-L6-v2 (22.7M)	Balances semantic accuracy and speed; suitable for CPU-based retrieval.	all-mpnet-base-v2 (110M): higher accuracy but significantly slower.
Vector database	FAISS (CPU)	Efficient similarity search with low memory and no network overhead.	Pinecone/qdrant: introduces latency and additional cost.
Chunking strategy	256–512 tokens (15% overlap)	Maintains contextual continuity while preserving retrieval precision.	Smaller chunks lose context; larger chunks reduce retrieval accuracy.
Generative model	DistilGPT-2 (82M)	Lightweight and CPU-deployable; sufficient for grounded response generation.	GPT-2 XL/GPT-3.5: higher cost and computational requirements.
Orchestration	LangChain	Modular pipeline design enabling flexible integration of RAG components.	Custom pipelines increase complexity without significant benefit.

## 4 Results and discussion

For evaluation, a total of 450 queries were selected from the dataset to ensure a comprehensive and statistically reliable assessment of system performance. The queries were categorized into three types based on complexity: (1) simple queries, (2) complex queries, and (3) compound queries. From each category, 150 queries were randomly selected, ensuring balanced representation across varying levels of reasoning difficulty.

Simple queries involve direct factual retrieval, complex queries require contextual understanding, and compound queries involve multi-step reasoning or multiple conditions. This categorization enables a structured evaluation of the system's ability to handle diverse query complexities. Each query was evaluated based on relevance and factual accuracy, following the metrics. The evaluation results demonstrate that the proposed system performs consistently across different query types, effectively handling both straightforward factual queries and more complex reasoning tasks.

The evaluation focuses on three key aspects: relevance, factual accuracy, and computational efficiency. Response relevance was assessed through human evaluation using a five-point Likert scale, while factual accuracy was verified against official Lok Sabha records. In addition, system efficiency was measured in terms of end-to-end inference latency on standard CPU hardware.

### 4.1 Cross-period generalization analysis

To evaluate the robustness and generalization capability of the proposed system, additional experiments were conducted using parliamentary data from multiple time periods, including 2021–2022, 2022–2023, and 2023–2024 (17th Lok Sabha).

For each time period, the same experimental setup was maintained, including identical preprocessing steps, embedding model, retrieval configuration (FAISS with top-*k* = 3), and generation model (DistilGPT-2). A balanced set of queries across simple, complex, and compound categories was used for evaluation.

The results indicate that the system demonstrates consistent performance across different time periods, with only marginal variations in both factual accuracy and response relevance. Similarly, the average inference latency remains stable across datasets, with negligible differences observed between time periods.

These findings suggest that the proposed retrieval-augmented framework is robust to temporal variations in parliamentary data and can generalize effectively across multiple legislative sessions without requiring retraining. This further supports the suitability of the system for real-world deployment in dynamic and continuously evolving parliamentary environments.

### 4.2 Category-wise performance analysis

To provide deeper insight into system performance, results are analyzed separately for each query category. Representative examples from each category are presented in Table 4.

**Table 4:** Representative examples across different query categories.

Query type	Question	Generated answer
Simple query	What is the objective of the Digital Personal Data Protection Bill?	The bill aims to regulate the processing of personal data to ensure privacy and accountability.
Complex query	How does the government justify the implementation of the new agricultural policy?	The policy is justified based on improving farmer income, increasing productivity, and ensuring sustainable agricultural practices, as discussed in parliamentary debates.
Compound query	What are the key provisions of the bill and who introduced it in Parliament?	The bill includes provisions related to data protection and was introduced by the Minister of Electronics and Information Technology.

The system demonstrates strong performance across all query types, with higher accuracy observed for simple queries and slightly lower performance for compound queries due to increased reasoning complexity. The quantitative results are summarized in Table 5.

**Table 5:** Category-wise performance of the proposed system (N = 450 queries).

Query type	Relevance score (1–5)	Factual accuracy (%)
Simple queries	4.8	96
Complex queries	4.5	93
Compound queries	4.2	90

The evaluation metrics used to assess system performance are summarized in Table 6.

**Table 6:** Evaluation metrics used for system performance assessment.

Metric	Description
Response relevance	Human evaluators rate each generated response using a five-point Likert scale (1: Irrelevant, 5: Highly Relevant), based on how accurately and completely it addresses the user's parliamentary query.
Factual accuracy	Binary metric (0/1) indicating whether all facts, dates, and references in the response are consistent with official Lok Sabha records.
Inference latency	Average time (in milliseconds) required for the complete pipeline, including retrieval and response generation, measured on a standard CPU (Intel Xeon E5-2680 v4).

### 4.3 Human evaluation protocol

To rigorously assess the quality of the synthesized responses, a human evaluation study was conducted on a total of 450 queries, evenly distributed across simple, complex, and compound categories. The evaluation panel consisted of five annotators with dual expertise in Indian parliamentary discourse and natural language processing. This combination ensured that responses were evaluated for both factual correctness and technical synthesis quality.

Each query-response pair was independently assessed by two annotators using a double-blind protocol to eliminate model-specific bias. Factual accuracy was verified against official Lok Sabha records, while relevance was rated on a five-point Likert scale (1: Irrelevant, 5: Highly Relevant). Annotators were instructed to prioritize completeness, contextual alignment, and formal parliamentary tone. In instances of rating disagreement, a third senior annotator reviewed the response to resolve the final rating through consensus. Inter-annotator agreement for the relevance metrics was quantified using Weighted Cohen's Kappa. The system obtained a Kappa score of 0.90, indicating a high level of consistency and reliability in the human assessment process across all query complexities.

### 4.4 Response relevance analysis

To evaluate the contextual relevance of the generated responses, experiments were conducted on a total of 450 queries sampled from the test dataset. The queries were evenly distributed across three categories: simple queries, complex queries, and compound queries, ensuring a balanced representation of varying reasoning complexities.

Each response was evaluated by a human panel using a five-point Likert scale (1: Irrelevant, 5: Highly Relevant), based on how accurately and completely the generated response addressed the user query. As shown in Table 7, the proposed RAG + DistilGPT-2 framework achieves the highest mean relevance score of 4.6, consistently outperforming the alternative lightweight transformer architectures under identical retrieval configurations.

**Table 7:** Response relevance comparison across transformer-based models (*N* = 450 queries).

Model architecture	Mean score	Median	Std. dev
RAG + FLAN-T5-Small	4.4	4.0	0.6
RAG + FLAN-T5-Base	4.5	4.0	0.5
**Proposed RAG + DistilGPT-2**	**4.6**	**5.0**	**0.4**

The results indicate that while all retrieval-augmented models benefit from external knowledge grounding, the RAG + DistilGPT-2 framework demonstrates superior synthesis of parliamentary context. While the RAG + FLAN-T5-Base model shows comparable relevance, it incurs significantly higher computational overhead, resulting in an average latency of 3,400 ms. In contrast, the proposed framework maintains superior relevance while being optimized for low-latency CPU deployment (1,800 ms), validating its practical suitability for real-time e-governance platforms.

### 4.5 Factual accuracy assessment

To rigorously assess the system's reliability, we employed a binary factual accuracy metric. A response was classified as Factually Consistent if all dates, names, and legislative provisions could be directly verified against official Lok Sabha records; otherwise, it was classified as Hallucinated/Inconsistent.

The standalone DistilGPT-2 model exhibits a higher hallucination rate, with only 80% of responses being factually consistent. This highlights the limitation of parametric models when used without external knowledge grounding. In contrast, the proposed RAG framework (RAG + DistilGPT-2) achieves 94% factual consistency, demonstrating the effectiveness of retrieval augmentation in grounding responses in authoritative parliamentary documents. A keyword search baseline serves as a reference for factual consistency, although it lacks the ability to generate coherent natural language responses.

**Figure 5:**
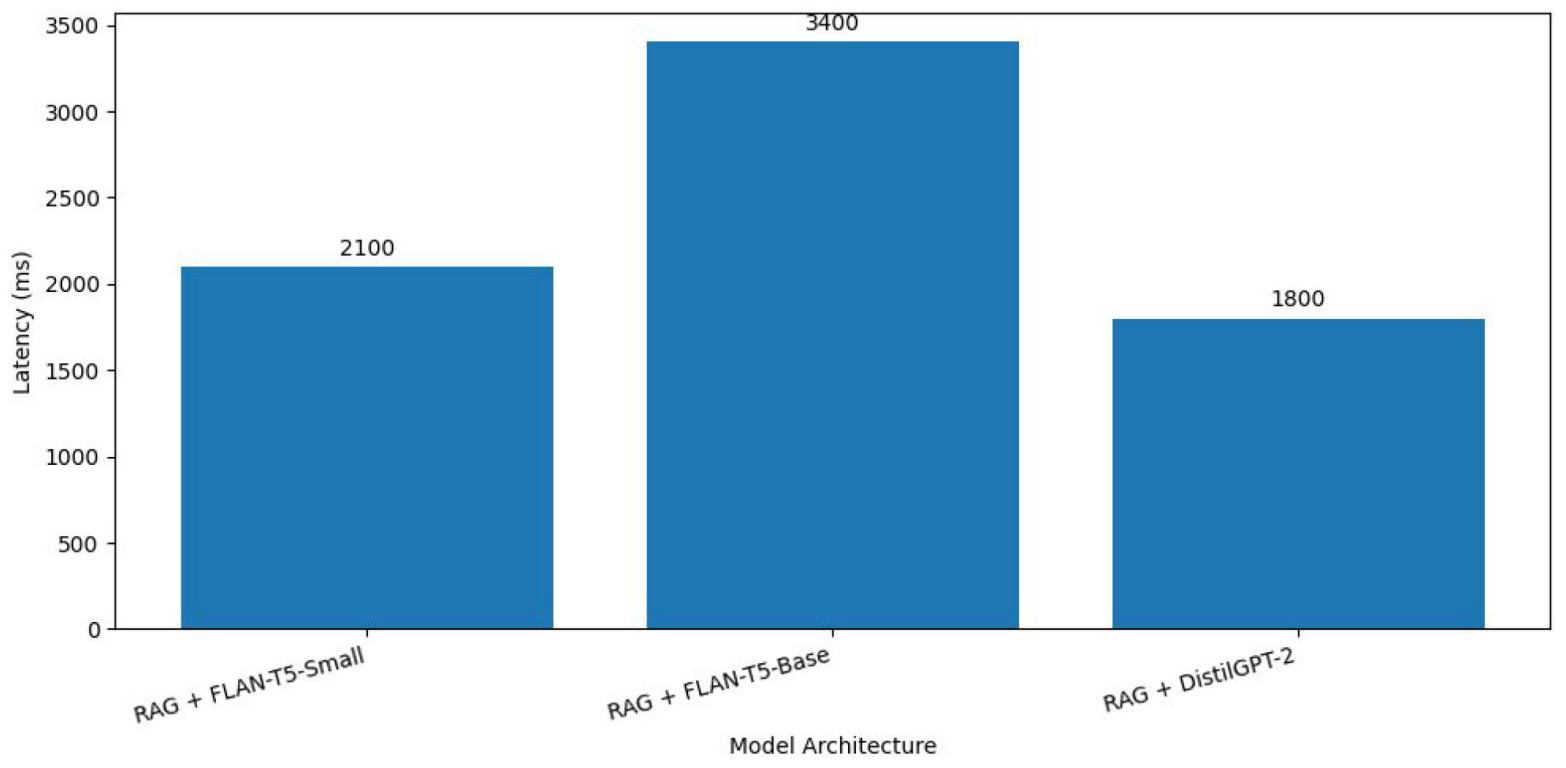
Inference latency comparison across evaluated models.

To further analyze system efficiency, inference latency was measured across the same set of 450 evaluation queries. Figure 5 illustrates the average end-to-end latency of the proposed system. A conventional GPT-2 XL based RAG system exhibits an average response time of 3,600 ms per query. This corresponds to an approximate three-fold improvement in inference speed, demonstrating the effectiveness of model compression and lightweight architecture design for low-latency deployment in resource-constrained environments.

### 4.6 Top-*k* retrieval ablation study

To justify the selection of the retrieval size (*k*), we conducted an ablation study by varying the number of retrieved text chunks. The evaluation was performed on a total of 450 queries, evenly distributed across simple, complex, and compound query categories, measuring both factual accuracy and inference latency.

**Figure 6:**
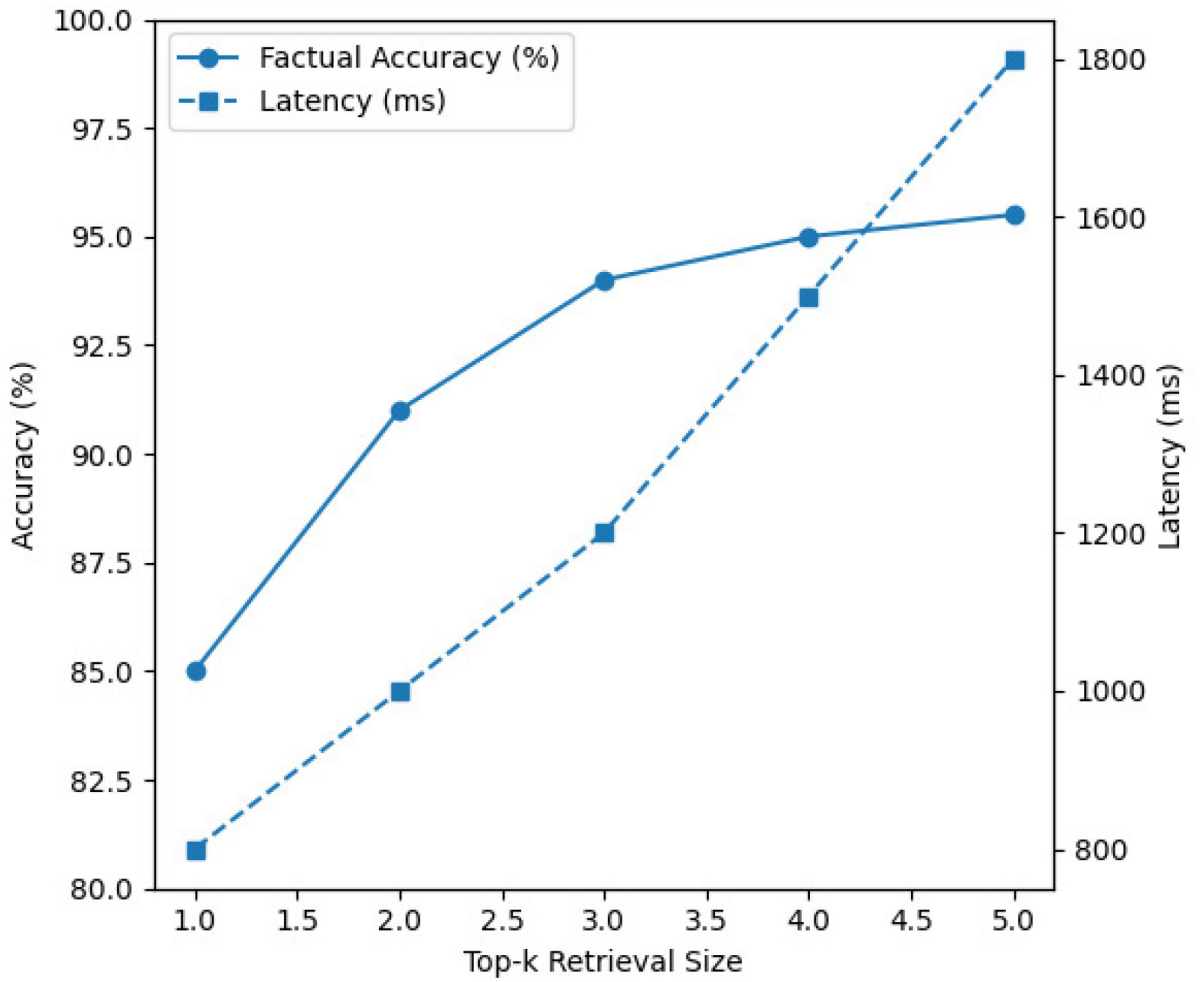
Effect of top-*k* retrieval size on accuracy and latency.

As shown in Figure 6, retrieving a single chunk (*k* = 1) often provides insufficient contextual information, resulting in lower factual accuracy. Increasing the retrieval size to *k* = 3 significantly improves response quality by incorporating additional relevant context.

However, further increasing the value of *k* beyond three leads to only marginal improvements in accuracy, while causing a near-linear increase in inference latency due to the larger input context processed by the generator. These results indicate that *k* = 3 provides an effective balance between accuracy and computational efficiency. Accordingly, this value was selected for all subsequent experiments in the proposed RAG framework.

### 4.7 Error analysis

To better understand system limitations, we analyze cases where the model fails to produce correct responses. The observed errors are categorized in Table 8.

**Table 8:** Common error types observed in the system.

Error type	Description
Incomplete retrieval context	Relevant documents are not retrieved within the top-*k* results, leading to partial or missing answers.
Multi-step reasoning errors	Compound queries requiring multiple reasoning steps result in incomplete responses.
Ambiguous queries	Vague or underspecified queries lead to responses that are relevant but not fully precise.
Numerical and entity errors	Occasional inaccuracies in numerical values or entity attribution due to similar references.

**Figure 7:**
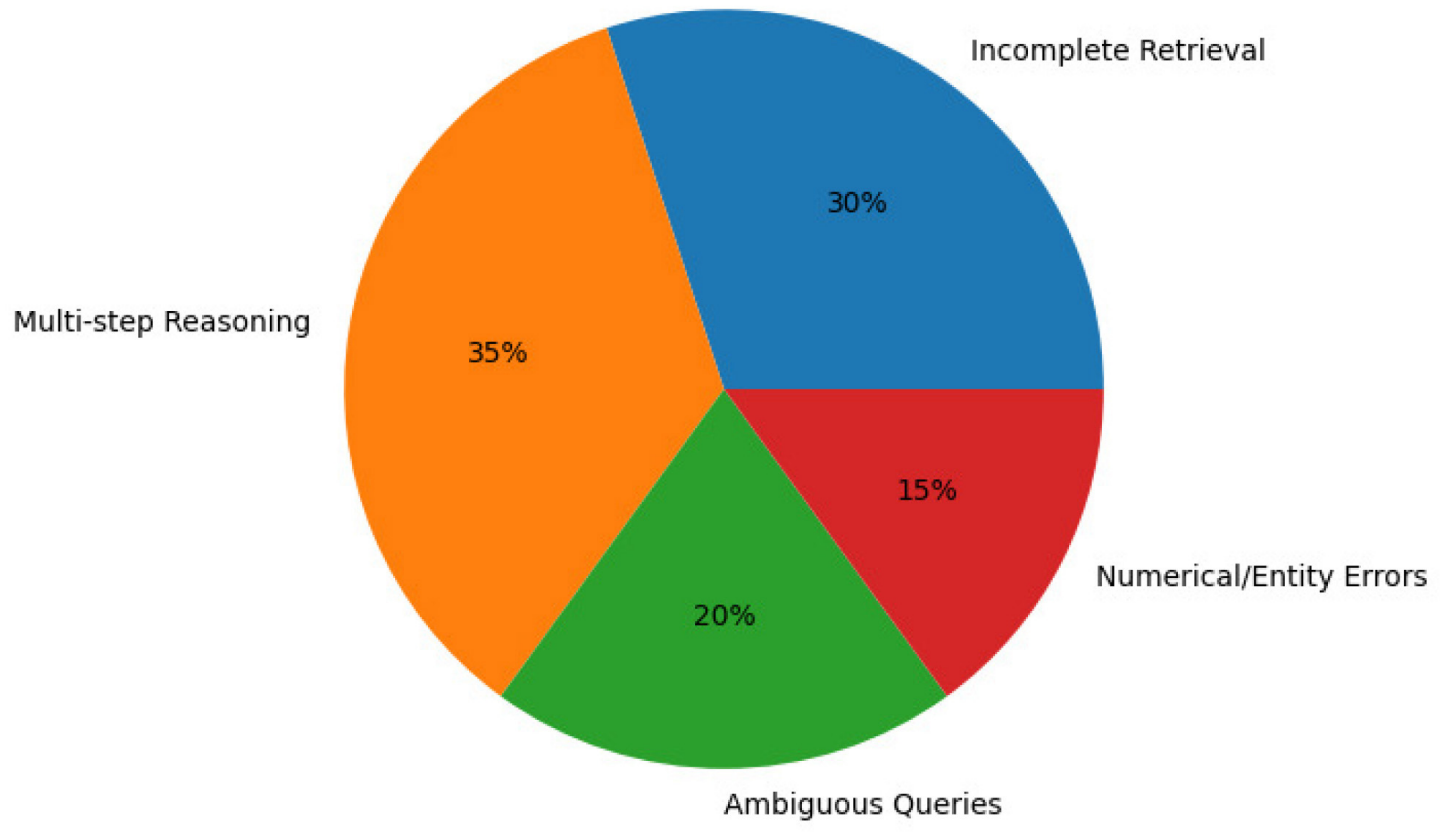
Distribution of error types across evaluation dataset.

Figure 7 further illustrates the distribution of these error types across the evaluation dataset.

The analysis reveals that errors primarily occur in scenarios involving multi-step reasoning and incomplete retrieval context. While retrieval augmentation significantly improves factual grounding, system performance remains dependent on retrieval quality and query complexity. These findings highlight opportunities for improving retrieval ranking and enabling more robust multi-hop reasoning in future work.

### 4.8 Qualitative analysis: handling hallucinations

A qualitative comparison highlights the impact of retrieval augmentation in mitigating hallucinations. Table 9 presents representative examples comparing the baseline generative model with the proposed system. The examples are selected from the evaluation dataset, covering different query categories to illustrate system behavior across varying levels of complexity. The baseline model frequently generates plausible but factually incorrect details due to reliance on parametric memory alone. In contrast, the proposed system produces responses grounded in retrieved parliamentary text, resulting in substantially improved factual consistency and reliability.

**Table 9:** Qualitative comparison of responses.

Query	DistilGPT-2 (without RAG)	Proposed RAG + DistilGPT-2
What is the penalty for data breach under the new Bill?	The penalty is set at $500 as per the 2010 regulations.	As per the Digital Personal Data Protection Bill, 2023, penalties for significant data breaches may extend up to 250 crore.
Who introduced the Jan Vishwas Bill?	The bill was introduced by the Prime Minister in 2018.	The Jan Vishwas (Amendment of Provisions) Bill was introduced by Shri Piyush Goyal, Minister of Commerce and Industry.

### 4.9 Semantic similarity distribution analysis

To assess the effectiveness of the semantic retriever, we analyze the distribution of cosine similarity scores between user queries and the retrieved parliamentary text chunks. A well-functioning retriever should consistently return documents with high semantic similarity, whereas a flat or uniform distribution would indicate weak or near-random retrieval behavior.

The distribution is highly skewed toward higher similarity values, with an average cosine similarity of 0.75. This concentration in higher similarity ranges indicates that the Sentence-BERT embedding model effectively captures semantic relationships between user queries and relevant Lok Sabha documents. Furthermore, the observed distribution demonstrates that the FAISS-based retrieval mechanism consistently returns contextually relevant passages, which directly contributes to improved factual accuracy and response relevance in the downstream generation process.

**Figure 8:**
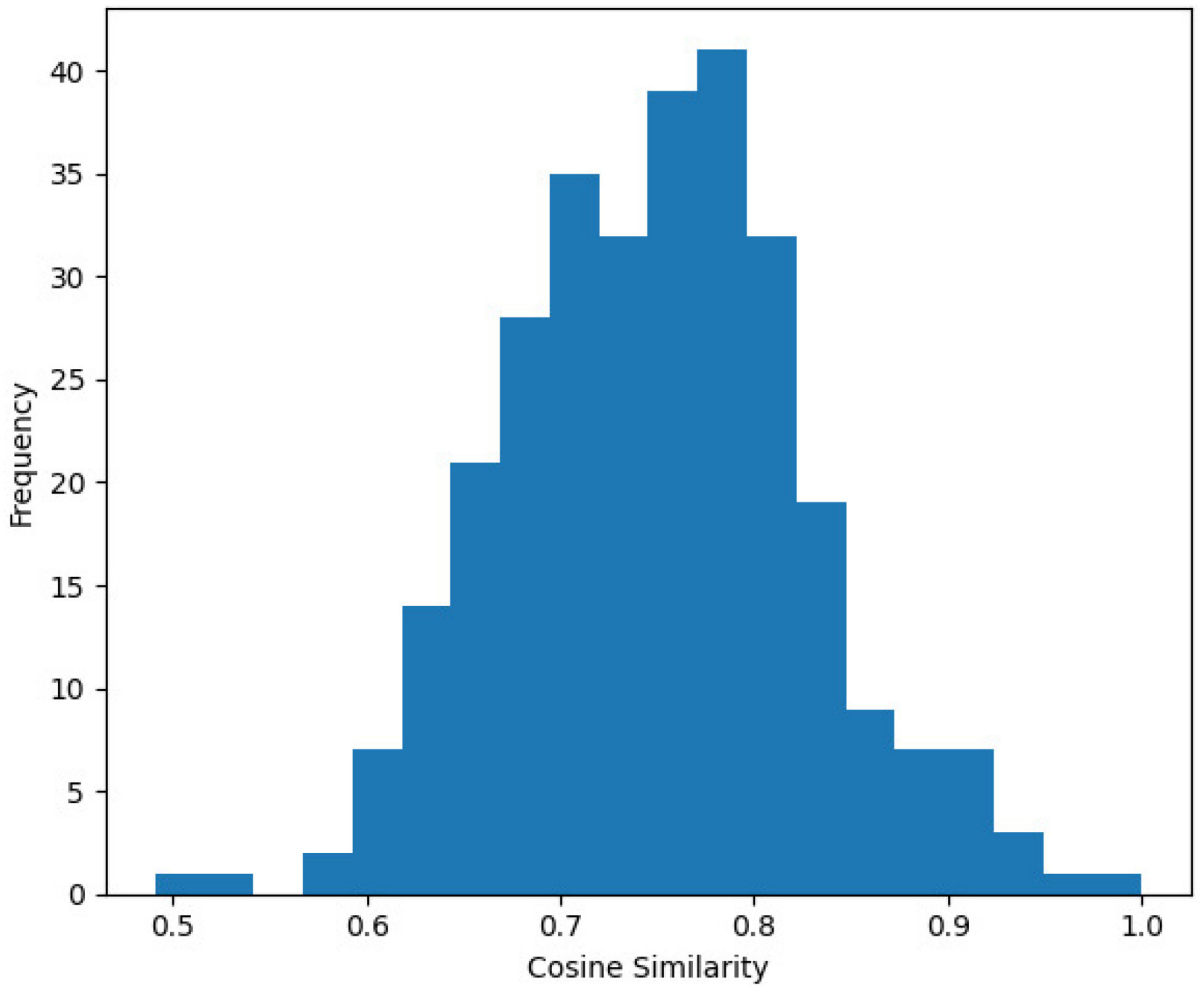
Distribution of semantic similarity scores for retrieved text chunks.

Figure 8 illustrates the distribution of cosine similarity scores for the top-*k* retrieved chunks across the evaluation dataset consisting of 450 queries, evenly distributed across simple, complex, and compound categories.

### 4.10 Quantitative performance comparison

The effectiveness of the proposed framework was evaluated through comparison with lightweight transformer baselines designed for resource-constrained deployment, namely FLAN-T5-Small and FLAN-T5-Base. To maintain experimental consistency, all models were assessed under the same retrieval configuration using the identical FAISS index, embedding model, and top-*k* retrieved parliamentary passages as grounded context. The comparative results are summarized in Table 10.

**Table 10:** Comparative performance of retrieval-augmented models.

Model architecture	Relevance score	Factual accuracy (%)	Latency (ms)
RAG + FLAN-T5-Small	4.4	91.5	2,100
RAG + FLAN-T5-Base	4.5	93.9	3,400
RAG + DistilGPT-2	4.6	94.0	1,800

The results indicate that retrieval augmentation consistently improves factual grounding across all evaluated models. Nevertheless, clear differences are observed in response quality and computational efficiency.

RAG + FLAN-T5-Small achieved a relevance score of 4.4 and factual accuracy of 91.5%. While the model benefits from external retrieval evidence, its comparatively lower performance suggests limited capacity for handling compound parliamentary queries and generating sufficiently detailed responses.

RAG + FLAN-T5-Base improved performance to a relevance score of 4.5 and factual accuracy of 93.9%, indicating stronger contextual synthesis and better answer precision. However, the model required the highest average latency of 3,400 ms, reflecting greater encoder–decoder computational overhead and reduced suitability for low-latency CPU deployment.

The proposed RAG + DistilGPT-2 framework achieved the best overall performance, with the highest relevance score of 4.6, factual accuracy of 94.0%, and the lowest latency of 1,800 ms. These findings demonstrate that the proposed framework provides the most effective trade-off between answer quality and computational efficiency. By combining lightweight generation with retrieval-based grounding, the system delivers accurate responses with faster inference, making it well suited for real-time parliamentary question answering in resource-constrained public-sector environments.

### 4.11 Computational efficiency

To evaluate computational efficiency, inference latency was measured on standard CPU hardware without GPU acceleration across the same set of 450 evaluation queries. The proposed RAG + DistilGPT-2 framework was assessed using complete end-to-end processing time, including document retrieval, prompt construction, and response generation.

For comparative reference, inference latency was also measured for retrieval-augmented baseline models under identical CPU-only hardware conditions. Although instruction-tuned architectures such as FLAN-T5-Small and FLAN-T5-Base provide strong response quality, they incur higher latency due to encoder–decoder computational overhead and increased model complexity.

The comparative latency results are summarized in Table 11.

**Table 11:** Inference latency comparison of retrieval-augmented models.

Model architecture	Average latency (ms)
FLAN-T5-Small + RAG	2,100
FLAN-T5-Base + RAG	3,400
**RAG + DistilGPT-2**	**1,800**

The proposed RAG + DistilGPT-2 framework achieved the lowest average end-to-end latency of 1,800 ms per query, outperforming FLAN-T5-Small + RAG (2,100 ms) and FLAN-T5-Base + RAG (3,400 ms). These findings indicate that the proposed framework offers a favorable balance between response quality and computational efficiency, making it suitable for real-time deployment scenarios.

The estimated computational cost of the proposed system is summarized in Table 12.

**Table 12:** Estimated computational cost of the proposed system.

Component	Estimated cost
Hardware	CPU-only (Intel Xeon E5-2680 v4, 2.4 GHz), no GPU required
Inference latency	1,800 ms per query
Energy consumption	216 Joules per query (estimated using 120W CPU × 1.8 s)
Memory requirement	2–4 GB RAM for model and FAISS index
Deployment cost	Low-cost deployment without specialized hardware

The system demonstrates low latency, moderate memory usage, and low deployment cost, making it suitable for resource-constrained environments such as public-sector and e-governance applications. It should be noted that the reported energy consumption values are approximate estimates derived from hardware specifications rather than direct power measurements.

### 4.12 Energy efficiency analysis

To estimate the computational efficiency of the proposed system, energy consumption was approximated based on CPU power usage and measured inference latency. The system was evaluated on an Intel Xeon E5-2680 v4 processor with an average power consumption of approximately 120W under load. All reported latency and derived energy values correspond to complete end-to-end processing time, including retrieval and response generation.

Given an average end-to-end inference latency of 1.8 s per query, the energy consumption per query is estimated as:

$$\begin{array}{l} E=P\times t \end{array}$$ (1)

where *P* denotes power consumption (in watts) and *t* denotes execution time (in seconds). Substituting the measured values:

$$\begin{array}{l} E=120\times 1.8=216\text{Joules per query} \end{array}$$ (2)

Table 13 shows the estimated energy consumption of all evaluated models based on measured inference latency and CPU power usage. Although the proposed RAG-enhanced framework consumes more energy than standalone DistilGPT-2 due to retrieval overhead, it remains substantially more energy efficient than larger instruction-tuned baselines such as FLAN-T5-Small and FLAN-T5-Base while delivering significantly improved factual accuracy and relevance.

**Table 13:** Estimated energy consumption of retrieval-augmented models.

Model architecture	Latency (s)	Power (W)	Energy per query (J)
RAG + FLAN-T5-Small	2.1	120	252
RAG + FLAN-T5-Base	3.4	120	408
RAG + DistilGPT-2	1.8	120	216

## 5 Conclusion and future scope

### 5.1 Conclusion

This study presented a domain-specific retrieval-augmented conversational agent designed to improve public access to Indian parliamentary proceedings. By integrating a lightweight DistilGPT-2 model within a RAG framework, the proposed system addresses two major challenges in governance-oriented AI systems: hallucination in generative language models and the unstructured nature of parliamentary documents available in PDF format.

Experimental evaluation conducted on 450 queries spanning simple, complex, and compound categories demonstrates that the proposed approach achieves a factual accuracy of 94%, substantially outperforming the standalone DistilGPT-2 baseline (42%). In addition, the system achieves a high relevance score of 4.6 out of 5 while maintaining an average end-to-end inference latency of approximately 1,800 ms on standard CPU hardware.

These results indicate that reliable and efficient AI-driven solutions for e-governance can be realized without reliance on large-scale models or expensive computational infrastructure. By transforming static legislative records into an interactive and queryable knowledge base, this work contributes to broader goals of digital transparency, informed civic participation, and inclusive access to democratic institutions.

Overall, the findings demonstrate that combining domain-specific retrieval with lightweight generative models can effectively balance factual accuracy, computational efficiency, and practical deployability in real-world governance applications.

### 5.2 Future scope

Despite the promising results, this study has certain limitations. The dataset is restricted to a specific subset of Lok Sabha proceedings, and the evaluation is conducted on a finite set of queries, which may affect generalizability across broader legislative contexts. Future work will focus on expanding the dataset to include multi-year parliamentary records and incorporating additional legislative sources such as Rajya Sabha proceedings. Benchmarking against more recent instruction-tuned and encoder-decoder transformer models will also be explored to further assess the scalability and competitiveness of the proposed approach. While the present study focuses on lightweight standalone FLAN-T5 baselines vs. the proposed RAG-based framework, future work will investigate retrieval-augmented FLAN-T5 variants to enable more architecture-matched comparisons.

Additional enhancements include extending the evaluation framework, integrating multilingual capabilities to support regional Indian languages, and developing voice-based interfaces to improve accessibility for diverse user groups. Furthermore, while the proposed system emphasizes computational efficiency, energy consumption and carbon footprint were estimated rather than directly measured in this study. Future work will involve detailed system-level power profiling and environmental impact assessment to provide a more comprehensive evaluation of deployment sustainability.

## Data Availability

The original contributions presented in the study are included in the article, further inquiries can be directed to the corresponding author.
